# Bio-Inspired Blade Cascades: Numerical Predictions Versus Experimental Measurements

**DOI:** 10.3390/biomimetics11030199

**Published:** 2026-03-09

**Authors:** Andrei-George Totu, Daniel-Eugeniu Crunțeanu, Dragoș Isvoranu

**Affiliations:** 1 “Elie Carafoli” Department of Aerospace Science, Polizu Campus, National University of Science and Technology POLITEHNICA Bucharest, Splaiul Independenței 313, 060042 Bucharest, Romania; daniel.crunteanu@upb.ro; 2 Gas Turbines COMOTI, National Research and Development Institute, 220D Iuliu Maniu, 061126 Bucharest, Romania

**Keywords:** bio-inspired, serrations, noise, blade cascade, spectra, SPL (sound pressure level)

## Abstract

This work presents a numerical–experimental validation of aeroacoustic predictions for bio-inspired leading edge serrated blade cascades. Transient simulations were carried out on a four-blade cascade using several turbulence modeling strategies commonly applied in broadband noise analysis—Spalart–Allmaras (SA), k−ω SST, k−ε, Scale-Adaptive Simulation (SAS), and Large Eddy Simulation (LES)—for assessing their capability to reproduce measured spectra. Multiple timestep resolutions were tested to ensure temporal accuracy. The comparison indicates that below 900 Hz, interaction noise is difficult to evaluate for such applications, whereas in the range from 0.9 to 5 kHz the turbulent jet–blade interaction is clearly captured. In the low-frequency regime (<1 kHz), the SA, SAS, and k−ω SST models exhibit similar behavior, while at higher frequencies SAS provides the closest agreement with experimental results, albeit with a slight tendency to overestimate at the upper end of the spectrum. LES demonstrates a satisfactory performance in reproducing the baseline response. The validation of numerical simulations with experimental results has been achieved, and a complex analysis using pressure measurements on the blade surface for a four-blade cascade configuration shows that turbulent formations lose their coherence quite significantly across several frequency bands. Overall, the results confirm that numerical simulations can reproduce the dominant experimental trends, while emphasizing the model-dependent trade-offs in predicting the acoustic benefits of bio-inspired leading edge serrations.

## 1. Introduction

Noise emissions from turbomachinery and compact aerodynamic devices remain a critical barrier to environmental compliance and societal acceptance. Stricter community noise limits and increasingly ambitious sustainability targets push designers toward solutions that reduce both tonal and broadband components [1,2] without compromising aerodynamic efficiency, structural integrity, or manufacturability [3,4,5]. Within this context, cascades of blades operating under turbulent inflow—typical of rotor–stator interactions, fans, compressors, or mixer–ejector configurations—are recurrent sources of broadband noise driven by unsteady loading and edge–turbulence interactions [5,6,7,8,9,10].

Conventional noise mitigation strategies span geometric and materials-based treatments, such as sweep and lean, skew, trailing edge thickness minimization, porous or compliant surfaces, and acoustic liners, as well as active flow control approaches based on periodic blowing or plasma actuators [8,11,12,13,14,15]. While effective in targeted regimes, these solutions often come with trade-offs, including added weight and complexity, efficiency penalties, narrowband effectiveness, or sensitivity to off-design operation. As a result, attention has shifted toward bio-inspired concepts that rely on flow physics mechanisms observed in nature [4,13,16,17,18,19] and aim for broadband efficacy with minimal actuation or maintenance overhead.

**Figure 1:**
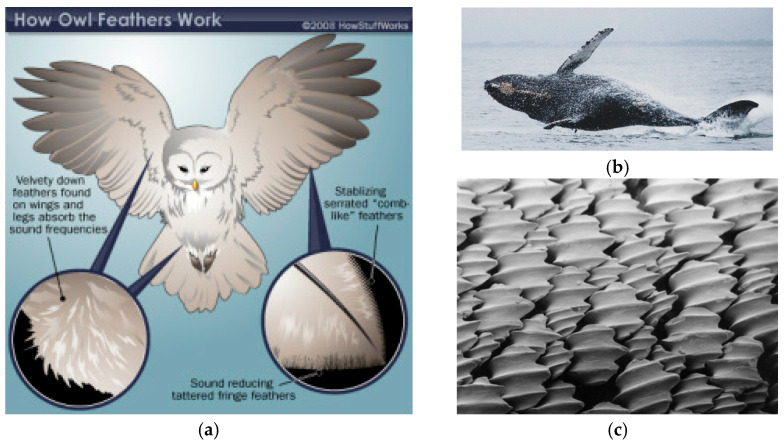
Adaptations identified in nature as potential inspiration for designing new blades: (**a**) owl adaptation [28]; (**b**) humpback whale tubercles [29]; (**c**) shark skin riblets [30].

Several bio-inspired motifs have contributed to aerodynamic and aeroacoustic design. Owl-inspired trailing edge serrations [20,21] disrupt spanwise coherence and reduce scattering efficiency at the trailing edge. Humpback whale leading edge tubercles [22,23,24] modulate stall progression and lift distribution. Shark skin riblets [25,26,27] reduce drag through streamwise alignment and near-wall vortex management. Figure 1 shows adaptations from nature that can be considered as a starting point for the design of quieter blades/stages. Building on these ideas, leading edge serrations have emerged as a promising option for mitigating inflow–edge interaction noise. In silent flight, owls rely on a multi-element edge treatment system: high amplitude, small pitch, comb-like structures near the leading edge, together with downstream micro-structures, reduce coherent scattering by promoting spanwise phase variation and broadband decorrelation of surface pressure fluctuations. From an engineering standpoint, leading edge serrations can be interpreted as a “manufacturable abstraction” of this strategy, aiming to reduce the spanwise coherence of unsteady loading before it is converted into noise. While the serration shape used in many studies (including the present one) may appear conventional, the biomimetic aspect is primarily functional rather than geometric. In practice, the spanwise-varying phase decorrelates the impingement of turbulent eddies, redistributes the relevant wavenumber content, and weakens the coherent unsteady forces responsible for sound generation. In cascades, where a turbulent jet/wake or any sort of incoming turbulence interacts with the blade row, this decorrelation is particularly valuable because it can limit the constructive build-up of unsteady loading across adjacent passages.

The advantages of leading edge serrations include their broadband noise potential attenuation, purely passive operation, and compatibility with existing cascade architectures. Nevertheless, limitations must be acknowledged. Aerodynamic penalties may arise from increased wetted area and local curvature, which affect loss coefficients and intended flow deflection [31]. Sensitivity to Reynolds number and inflow properties, such as turbulence intensity and integral length scale, can shift the effective frequency band [32,33]. Structural and manufacturing complexity is expected to increase with serration amplitude and wavelength, while unintended spectral features may emerge when serration spacing interacts with coherent structures in the inflow. Successful application therefore requires a careful balance between acoustic benefit and aerodynamic cost. From a biomimetics perspective, this balance is not merely a design compromise but a functional translation constraint: the geometry must remain manufacturable and aerodynamically acceptable while still enforcing the measurable spanwise decorrelation of leading edge pressure fluctuations.

The transition from a straight-edged blade to a serrated leading edge is typically parameterized by serration amplitude (relative to chord), wavelength (relative to chord), and (local) blade skew, together with operational parameters such as Reynolds number, Mach number, incidence, turbulence intensity, and the ratio between incident turbulence length scale and serration wavelength. Prior studies suggest that the strongest decoherence benefits occur when incoming turbulence scales are commensurate with the serration wavelength, while far-field spectral trends tend to scale with Strouhal number [34,35]. These considerations provide motivation for systematic parametric exploration at a reduced scale before committing to full-scale implementation.

Performance assessment methods can be grouped into analytical models [8,25,36,37,38], numerical simulations [8,19,20,21,22,23,29,32,35,39,40], and experimental techniques [7,24,27,31,33,36]. Analytical and semi-empirical formulations for leading edge noise predict far-field spectra from wall pressure or inflow statistics and capture first-order parametric trends, while their serration-extended variants introduce spanwise phase modulation to represent decorrelation. These methods are computationally inexpensive but depend strongly on modeling assumptions. Computational approaches based on steady RANS provide reliable low-cost aerodynamics but lack fidelity for broadband spectra [14,19,29,41]. Unsteady approaches, such as URANS, SAS, DES, and LES, increasingly resolve the unsteady flow content responsible for noise radiation, with far-field prediction obtained through acoustic analogies such as Ffowcs Williams–Hawkings (FW-H). Accuracy depends on turbulence modeling, grid resolution near edges, numerical dissipation, and temporal resolution. On the experimental side, cascade wind-tunnel campaigns remain the benchmark for validation, combining steady and time-resolved pressure measurements, wake development, and far-field microphone data to quantify both aerodynamic performance and aeroacoustics output. In this context, reduced-scale testing represents a powerful and time-efficient path to evaluating serrated concepts. Results at laboratory scale can be regarded as satisfactory, and thus transferable to higher technology readiness levels, when they demonstrate consistent broadband noise reduction without unacceptable aerodynamic penalties and when they prove to be robust to variations in turbulence intensity, length scale, incidence, and Reynolds number, without introducing new adverse spectral features. When these conditions are met, small-scale findings can be scaled to full-size applications through similarity-based methods/algorithms, supported by complementary simulations or tests.

The present study addresses the numerical–experimental validation of bio-inspired leading edge serrated cascades. Reduced-scale experiments provide controlled conditions and a high spatial resolution, while a range of turbulence modeling strategies—Spalart–Allmaras, k−ω SST, k−ε, SAS, and LES (whose formulations are very well covered in [41,42,43])—are evaluated in transient simulations with varying timestep resolutions. The focus is on their capability to reproduce measured spectra and to capture the dominant mechanisms of jet–blade interaction noise. Comparisons with experiments indicate that interaction noise is negligible at low frequency and clearly captured at higher frequencies (over 2 kHz). While Spalart–Allmaras and k−ω SST reproduce the low-frequency regime, SAS achieves the closest overall agreement in the mid-to-high frequency range, albeit with slight overestimation, and LES satisfactorily reproduces the baseline response at a higher computational cost. These findings highlight model-dependent trade-offs, clarify temporal resolution requirements, and confirm the relevance of numerical simulations as predictive tools for the aeroacoustic benefits of bio-inspired leading edge serrations. Beyond far-field SPL reduction, serrations are expected to modify the spanwise pressure distribution (on the leading edge). To quantify this effect, spanwise coherence and the associated coherence length are introduced. This choice is directly aligned with the biomimetic design objective, since leading edge serrations are intended to reduce spanwise coherence as the primary pathway to broadband noise suppression. The present work is intended as a preliminary validation step for the CFD workflow, setting a certain level of confidence at a reduced scale before transferring the methodology to a more representative, near-full-scale compressor/turbine configuration.

## 2. Materials and Methods

### 2.1. Experimental Approach

**Figure 2:**
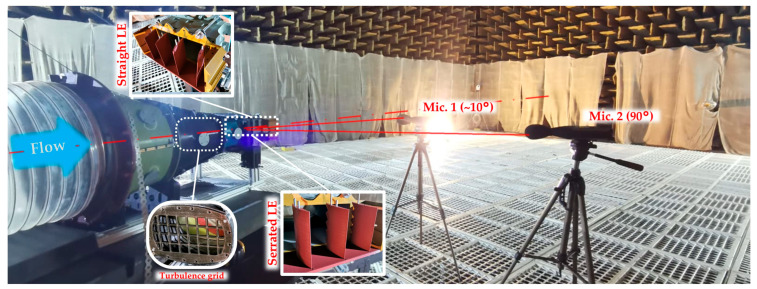
Experimental setup.

Experiments were conducted in an anechoic facility, designed to satisfy ISO 3745 [44] free-field conditions. The chamber has a volume of approximately 1200 m^3^ (15 m × 10 m × 8 m) with wall absorption ≈99% in the 150 Hz–20 kHz band [45]; the compressor was located outside the chamber and connected via a 20 m flexible duct to the in-room test section, arranged diagonally to minimize curvature of the supply line. Free-field conditions were ensured by placing the measurement devices sufficiently far from reflecting boundaries and by operating above the room’s effective cut-on frequency; the residual influence of reflections was minimized by the high absorption treatment and the diagonal arrangement of the rig. The measurement procedure follows the intent of ISO 3745 in providing an essentially free-field environment over a reflecting plane within the frequency range used for the present comparisons. Two Class 1 sound level meters (Acoem Fusion, Limonest, France) were used, positioned on a 1 m radius circular arc as in Figure 2. The quasi-axial microphone was placed at approximately 10° relative to the jet axis to remain in the acoustic far field while avoiding direct exposure to the high-velocity jet core and associated pressure “contamination”. The 90° microphone complements this view by capturing lateral radiation, which is typically more sensitive to loading noise directivity. Raw signals were sampled at 50 kHz. The dataset comprises two operating conditions: self-noise, with flow generated only by the convergent nozzle, and interaction noise, in which a 3D-printed rectangular turbulence grid was inserted in the nozzle to intensify inflow fluctuations and promote jet–blade interaction. The 3D-printed turbulence grid had a nominal cell of 20 × 20 mm and 4.5 mm thickness and was placed ~300 mm upstream of the leading edge. The 3D-printed grid acts as a passive turbulence generator that increases turbulence intensity and sets an integral length scale upstream of the cascade, thereby reproducing a representative interaction noise inflow condition. This controlled inflow enables a consistent experimental reference for validating the turbulence model dependence of the predicted spectra, particularly in the 2–5 kHz band where turbulence–leading edge interaction dominates. Data were processed in dBFa (ACOEM) using FFT in 10–20,000 Hz with 1.25 Hz resolution, Hanning windowing, 50% overlap, and RMS estimation.

The cascade comprised four vanes mounted downstream of the convergent nozzle. Two geometries were assessed at an incidence angle of 15°: a baseline blade with a straight leading edge and a bio-inspired blade with triangular leading edge serrations. The serrations were rounded-tip cutouts with amplitude equal to 20% of the chord and 15 mm pitch; other geometric parameters, including chord and vane spacing, followed the configuration described in [45].

The test used as experimental data in the comparative analyses performed in the manuscript is part of a larger testing campaign where both leading edge and trailing edge serrations were tested. Trailing edge serrations tend to work well in reducing self-noise, while those placed on the leading edge are effective in reducing interaction noise. Before each type of geometry, the consistency of the reference blade spectra (for all AoAs analyzed) was checked to ensure repeatability. On the aerodynamic side, an L-shaped Pitot tube was used to reconfirm that the fluid velocity is consistent (the air source only has an on–off function, without speed variation). The microphone signals were recorded for approximately 30 s for each test condition, and the acoustic results were obtained from time-averaged spectral estimates to improve spectral convergence and reduce the variance (finite sample variability) of the broadband jet–blade interaction noise [45].

### 2.2. Numerical Approach

**Figure 3:**
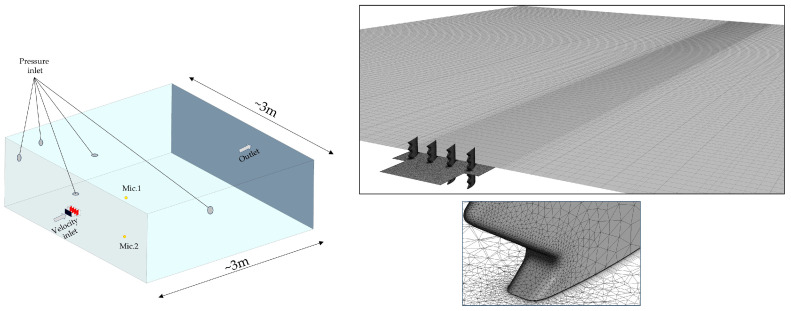
Computational domain and mesh.

The computational domain mirrored the experimental arrangement and was divided into two regions. The near-field region (77 mm × 75 mm inlet zone and 62 mm × 220 mm box around blades) was for resolving the cascade and the inlet zone used a predominantly structured mesh (Figure 3) around solid boundaries—the four blades, the side walls, and the top and bottom of the first domain (the one containing the blades)—with near-wall spacing chosen to achieve y+ ≈ 1 (a first layer thickness of ~10^−5^ m). A velocity inlet boundary condition was used for the vane section upstream area with a mean speed of 50 m/s, turbulence intensity Tu = 10%, and integral length scale Λ = 2.5 mm, representing the interaction noise condition produced experimentally by the physical grid. The outer region extended to around 3 m in length and 3.22 m width to include virtual microphone points coincident with the experimental sensor locations (1 m radius at 10° and 90° relative to the longitudinal axis of the test setup). For a medium size mesh (close to 4 M elements), near-blade domain elements are limited to a maximum size of ~1.25·10^−4^ m and for the outer go up to ~2.5·10^−2^ m (global mesh size). Far-field boundaries (set as atmospheric pressure inlets on the side walls, as well as a pressure outlet on the outflow area as shown in Figure 3) were set non-reflecting to avoid acoustic contamination of the spectra. Five turbulence modeling strategies commonly used for broadband aeroacoustics were evaluated on both geometries: Spalart–Allmaras (SA), k−ω SST, k−ε, Scale-Adaptive Simulation (SAS), and Large Eddy Simulation (LES). Transient solutions were initialized from a statistically steady state obtained with a 1 ms physical time step; stabilization was verified by monitoring the temporal evolution of *p*_rms_ and *dp*/*dt* until stationarity. The unsteady runs then proceeded with a time step *Δt* = 10^−5^ s for 1800–2000 time steps and up to 20 inner iterations per step (to ensure that spectral roll-off and peak locations were not artifacts of under-resolved unsteadiness).

The FW-H formulation described in [46] and Equation (1) were used for noise prediction:

(1) $$\frac{\partial^{2}p^{\prime }}{\partial t^{2}}-c_{0}^{2}\nabla^{2}p^{\prime }=c_{0}^{2}\frac{\partial^{2}}{\partial x_{i}\partial x_{j}}\left[T_{ij}H\left(f\right)\right]-c_{0}^{2}\frac{\partial }{\partial x_{i}}\left\{\left[P_{ij}n_{j}+\rho u_{i}\left(u_{n}-v_{n}\right)\right]\delta \left(f\right)\right\}+\frac{\partial }{\partial t}\left[{\rho \;c}_{0}^{2}\left(u_{n}-v_{n}\right)\;\delta \left(f\right)\right]$$

with H(f)—the Heaviside function (unity outside the body), δ(f)—the Dirac distribution on the moving surface S, outward normal $n_{i}$, flow velocity $u_{i}$ (with $u_{n}=u_{i}n_{i}$) and surface normal speed $v_{n}$. Compressive and Lighthill stress tensors are written in Equation (2):

(2) $$P_{ij}=p\delta_{ij}+\rho u_{i}u_{j}-\tau_{ij},\;\;\;\;T_{ij}=P_{ij}-c_{0}^{2}\rho \delta_{ij}$$

where $\tau_{ij}$ is the viscous stress tensor. For a fixed, impermeable surface $\left(v_{n}=u_{n}=0\;\mathrm{on}\;S\right)$, the thickness (monopole) term in Equation (1) vanishes and the dipole reduces to $-\partial_{x_{i}}[p\;n_{i}\;\delta (f)]$. ANSYS Fluent implements the FW-H post-processing in Farassat’s Formulation 1A (time-domain integrals—surface formulation [19,46]), evaluating the time-domain surface integrals in Equations (3)–(5) at specified observer points. For an observer at x, let R=x−y, $R=\left|R\right|$, r=R/R, $M=U/c_{0}$ the material Mach vector of the surface, $M_{r}=M\cdot r$, and define the loading vector $L_{i}=P_{ij}n_{j}$ with $L_{r}=L_{i}r_{i}$. Using retarded time $\tau_{r}$ (all bracketed quantities evaluated at $\tau_{r}$), we can write

(3) $$p^{\prime }(x,t)={p_{T}}^{\prime }(x,t)+{p_{L}}^{\prime }(x,t)$$

(4) $$p_{T}^{\prime }\left(x,t\right)=\int_{S}{\left[\frac{\rho_{0}\left(v_{n}-u_{n}\right)}{4\pi R\left(1-M_{r}\right)}\right]}_{ret}dS$$

(5) $${p_{L}}^{\prime }(x,t)=\frac{\partial }{\partial t}\int_{S}{\left[\frac{L_{r}}{4\pi c_{0}R\left(1-M_{r}\right)}\right]}_{ret}dS+\int_{S}{\left[\frac{L_{r}}{4\pi R^{2}{\left(1-M_{r}\right)}^{2}}\right]}_{ret}dS$$

For rigid, impermeable surfaces $u_{n}=v_{n}=0$, the thickness term $p_{T}^{\prime }$ is zero and the radiation is purely loading noise. These relations correspond to the formulation in [22] and are consistent with the tensor definitions in Equation (2). Two source-surface options are available: impermeable (the solid blade and walls) and permeable (a control surface in the flow). In the impermeable case typically used here, the thickness term Equation (4) is zero and only the loading term Equation (5) contributes; quadrupole volume sources from Lighthill’s tensor are not included by the FW-H post-processor (they would require separate modeling). Practically, the workflow is: (i) select the FW-H surface(s) (blades/near-blade shroud), (ii) define observers at 1 m and 10°/90°, (iii) run the transient with *Δt* = 10^−5^ s, and store the surface data needed for Equation (5), (iv) let Fluent assemble *p*′(*t*) at each observer from the retarded-time integrals. Frequency-domain SPL is then obtained by FFT of *p*′(*t*) using the same windowing and overlap as the experiment. Statistical stationarity was assessed by monitoring pressure and pressure gradient signals at multiple locations until their mean and RMS values stabilized over several flow-through times. Given the inflow velocity and the streamwise extent of the computational domain, the incoming flow crossed more than 80% of the domain length prior to the start of unsteady data acquisition, which is consistent with accepted practices for establishing steady or statistically stationary conditions in cascade simulations.

### 2.3. Unsteady Derived Metrics

**Figure 4:**
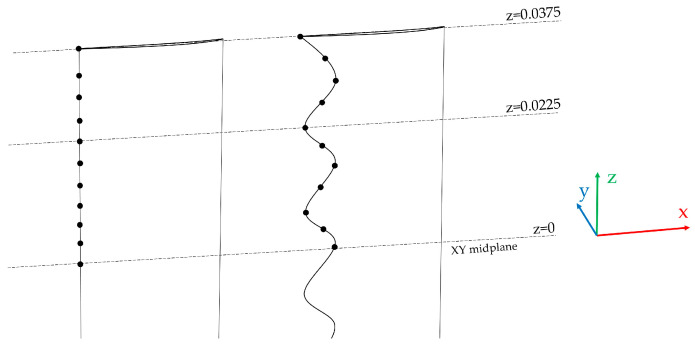
Leading edge points for baseline and serrated geometry.

To quantify the three-dimensional structure of the unsteady surface pressure field along the leading edge, we evaluated the magnitude-squared coherence of the pressure fluctuations measured at discrete spanwise locations. For each point *i*, as in Figure 4 (corresponding to the nearest surface mesh node at various z positions), the fluctuating component was obtained as in Equation (6), followed by a linear detrending to remove slow drift in the quasi-static pressure signal. The acquisition files report only the spanwise coordinate z, hence the present analysis quantifies coherence strictly as a function of *Δz* along the leading edge line. This is appropriate for a first-order “spanwise coherence” assessment, because the principal design intent of leading edge serrations is to modify interaction mechanisms distributed along the span. The spanwise direction corresponds to the blade height direction, while the streamwise direction is aligned with the incoming flow at the leading edge (as the pressure term from the fluctuating pressure coefficients [47]).

(6) $$p_{i\left(t\right)}^{\prime }=p_{i\left(t\right)}-\bar{p_{i\left(t\right)}}$$

The magnitude-squared coherence between two locations *i* and *j* was computed in the frequency domain as in [48] as follows:

(7) $$\gamma_{ij\left(f\right)}^{2}=\frac{{\left|G_{ij\left(f\right)}\right|}^{2}}{G_{ii\left(f\right)}\cdot \;G_{jj\left(f\right)}}$$

where $G_{ii\left(f\right)}$ and $G_{jj\left(f\right)}$ are auto power spectral densities (PSDs) and $G_{ij\left(f\right)}$ is the cross power spectral density (CPSD). Spectral estimates were obtained using Welch’s averaging (Hanning window, 50% overlap, NFFT = 512), consistent across baseline and serrated configurations to ensure comparability. Since the points were placed along the leading edge at known spanwise coordinates z (provided in the simulation related pressure variation export file), the separation for a pair is $\Delta z_{ij}=\left|z_{j}-z_{i}\right|$. To obtain relevant metrics, coherence was computed over user-defined frequency bands $\left[f1,\;f2\right]$ and over all pairs as follows:

(8) $$\bar{\gamma^{2}}(\Delta z;\;f1,\;f2)\;=\;median_{\left(i<j\right),\;\Delta z_{ij}=\Delta z}\;[\;median_{f\in \left[f1,f2\right]}\gamma_{ij}^{2}\left(f\right)]$$

A two-stage median aggregation is employed: first across frequency bins within each band to mitigate the effect of narrowband spectral spikes, and subsequently across all sensor pairs sharing the same spanwise separation *Δz*. This strategy provides increased robustness relative to single-reference coherence and prevents bias associated with a particular spanwise location. A compact length scale was extracted by fitting an exponential decay model, commonly used to represent spanwise decorrelation of wall pressure fluctuations. A compact length scale was extracted by fitting the following exponential decay model, commonly used to represent spanwise decorrelation of wall pressure fluctuations (as in [49] or [50]):

(9) $$\gamma^{2}\left(f,\;\Delta z\right)\approx \exp\left(-\frac{2\Delta z}{L_{z}\left(f\right)}\right)$$

For each frequency band, *L_z_* was estimated from all available $\left(\Delta z_{ij},\bar{{\gamma^{2}}_{ij}}\right)$ pairs via a least-squares slope through the origin after logarithmic linearization as follows:

(10) $$y\;=\;-\frac{1}{2}\cdot \ln\left(\gamma^{2}\right)\approx \;\frac{\Delta z}{L_{z}}\;\to \;L_{z}\;=\;{\left[\frac{{\Delta z}^{T}\;\cdot \;y}{{\Delta z}^{T}\;\cdot \;\Delta z}\right]}^{-1}$$

To prevent numerical artifacts, *γ*^2^ was clipped to the interval [10^−6^, 0.999999] prior to the logarithm. In addition, frequency bins where PSD energy was too low (e.g., more than 60 dB below the channel’s PSD maximum) were discarded to avoid coherence values dominated by numerical noise rather than physical correlation. The parameters defining the characteristic lengths of coherence are defined in various forms, with variants found in the literature that also take into account the parameters of the boundary layer (momentum or displacement thickness) [47,48,49,50,51].

## 3. Results

**Figure 5:**
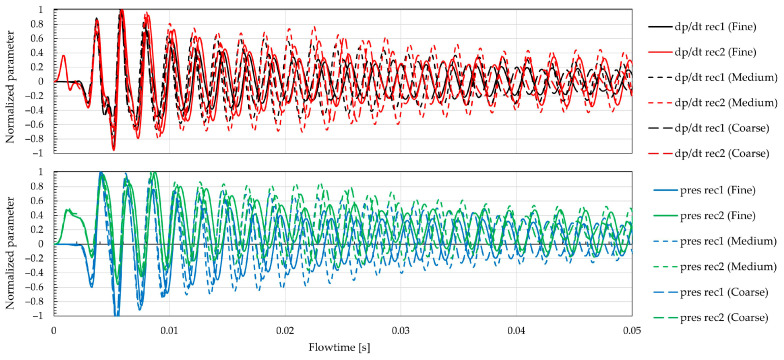
Steady solution and mesh independence (LES, *dt* = 10^−4^).

A grid convergence (mesh independence) study was performed by varying the mesh size from 0.68 M nodes and 1.71 M elements (coarse) to 5.18 M nodes and 13.4 M elements (cine) using the same setup (boundary conditions) as in Figure 3. An intermediate (medium) grid (2.12 M nodes, 4.11 M elements)—offering the best compromise between spectral fidelity and computational cost—was adopted for the turbulence model comparison. Figure 5 presents normalized time histories of the pressure time derivative *dp*/*dt* (top) and static pressure p (bottom) at the two numerical receivers (≈10° and 90°), from coarse to fine grids (the signals were normalized to the maximum value). After initialization, both records reach statistically stationary conditions and display periodic content that anticipates the fluid–structure interaction seen later. The phase and amplitude envelope are consistent across meshes, with modest amplitude differences—slightly larger at mic 1—suggesting that the near-observer unsteady loading is captured, while mesh effects are concentrated in the low–mid frequencies. These signals therefore indicate that the temporal resolution is adequate and that the serrated configuration promotes repeatable, spanwise-modulated fluctuations at the observers; the forthcoming SPL/PSD comparisons can thus focus on quantifying how turbulence models distort the sub-kilohertz region versus their mid-band alignment with the measurements.

Verification runs performed during mesh convergence checks (based on SPL/PSD) indicated consistent trends at both receivers: for mic1 (rec1), the coarse grid systematically overestimated the low–mid band, with a broad elevation up to 1 kHz, whereas the medium grid reproduced the experimental interaction noise spectra more faithfully over 1–5 kHz range; the fine grid did not deliver systematic improvements and introduced pronounced undulations and local peaks (notably near 1.5–3 kHz) absent from the measurements. For the lateral microphone, the coarse grid again overshot the 0.5–1 kHz region, the medium grid remained closest to the data across most of the band, and the fine grid tended to sit higher and comparatively flat with a notch near 2 kHz. These trends justify adopting the medium grid for turbulence model comparisons.

**Figure 6:**
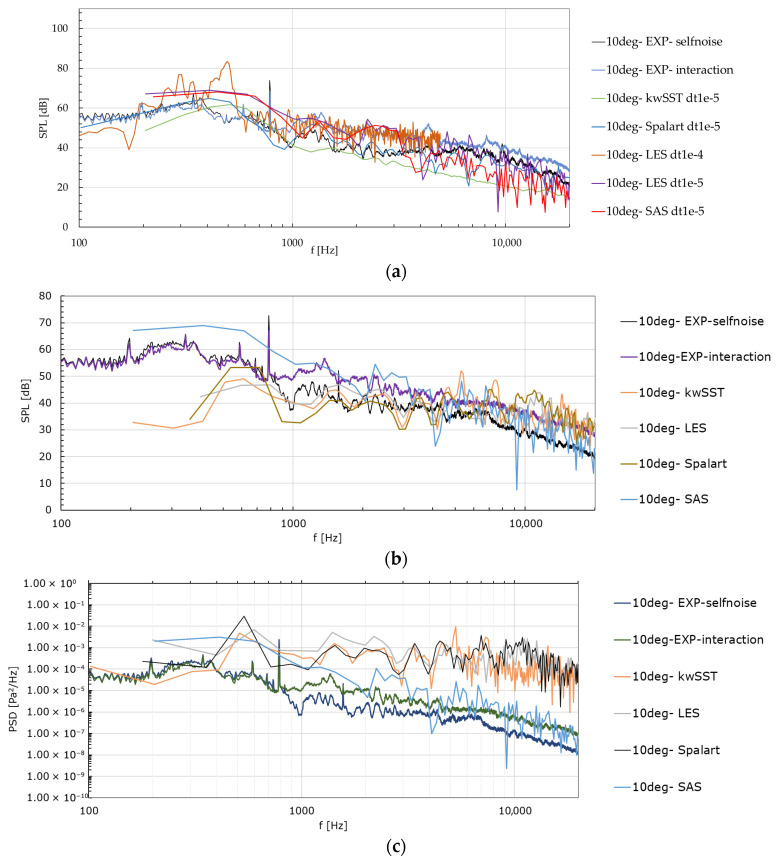

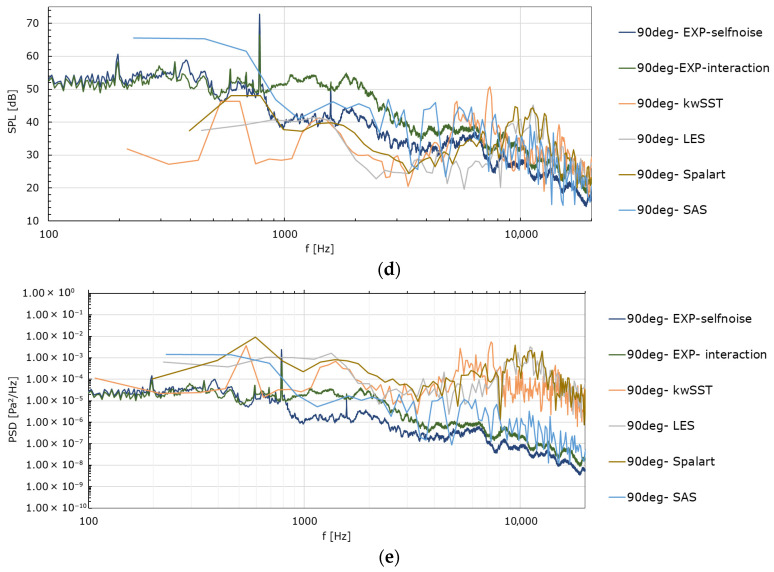
Comparative spectra: (**a**) various combinations on baseline geometry (10° direction); (**b**) various models on serrated geometry (10° direction, *dt* = 10^−5^ s); (**c**) PSD on serrated geometry (10° direction, *dt* = 10^−5^ s); (**d**) various models on serrated geometry (90° direction, *dt* = 10^−5^ s); (**e**) PSD on serrated geometry (90° direction, *dt* = 10^−5^ s).

The results for baseline and serrated configurations are presented in Figure 6 (run on Fluent 2021 R1). Multiple combinations of timestep sizes and turbulence models were tested, with the corresponding data extracted and processed to obtain the acoustic sound pressure level (SPL) spectrum. Unless otherwise indicated in the figure, the simulations used the medium mesh, were initialized from a stabilized solution (1 ms), and advanced for approximately 1500–1800 time steps. The SPL was derived by converting the numerically obtained PSD following the FFT. Overall, the model predictions are broadly consistent, with model-dependent features over certain frequency ranges—most notably at low frequencies (<1 kHz)—and close agreement in the band of interest (2–5 kHz), particularly for the SAS model, which is relatively inexpensive computationally.

**Figure 7:**
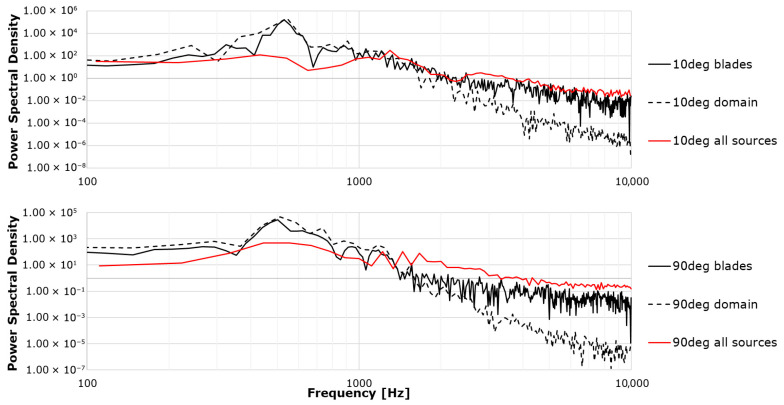
Acoustic sources’ influence.

Regarding the contributors to noise, a distinction can be made between the influence of the near-blade region (mainly dipole sources) and that of the outer domain (quadrupole sources corresponding to unbounded flow); the total perceived noise results from the combined effect of both. Solely to quantify the relative contribution of each, Figure 7 overlays the spectra computed for three acoustic source scenarios—near-blade domain, outer domain, and the combined case—sampled at the same receiver locations as in the preceding figures. For this analysis, the k−ω SST model was used with a time step of *Δt* = 10^−5^ s, and results were obtained after just over 500 time steps.

The PSD overlays (Figure 7) indicate that the near-blade region controls the spectrum across almost the entire band starting from the sub kiloHertz region. The outer domain contribution decays rapidly with frequency and becomes secondary as viscous dissipation suppresses wake-borne fluctuations (which was to be expected for a relatively low Reynolds numbers). The combined spectra essentially collapse onto the near-blade curves, for both angles, confirming that aeroacoustics content is dominated by the flow physics (such as separation, unsteady loading, and local edge interactions) in the immediate vicinity of the blades. This aligns with the observation that improving near-wall resolution (y+ ≈ 1) and controlling temporal accuracy in that region are the two ingredients responsible for high spectral fidelity.

**Figure 8:**
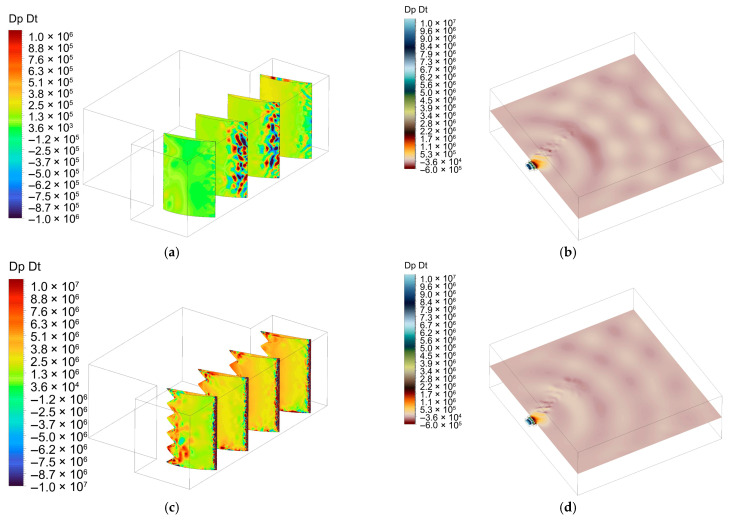
Comparative *dp*/*dt*, LES, *dt* = 10^−4^ s, t ≈ 50 ms: (**a**) baseline (blade distribution); (**b**) baseline (axial plane distribution); (**c**) serrations (blade distribution); (**d**) serrations (axial plane distribution).

Figure 8a–d provides complementary perspectives of the same unsteady loading indicator (*dp*/*dt*). The blade-attached views in Figure 8a,c highlight the spatial distribution of strong loading events along the leading edge, whereas the axial plane cuts in Figure 8b,d show how these events travel downstream too. Compared to the baseline case (Figure 8a,b), the serrated configuration (Figure 8c,d) exhibits a visibly shorter spanwise continuity and reduced streamwise persistence of high-amplitude *dp*/*dt* patches. This spatial fragmentation is consistent with serration-induced phase modulation at the leading edge, which weakens the coherent accumulation of unsteady forces and supports the spectral reductions observed in the interaction noise band.

**Figure 9:**
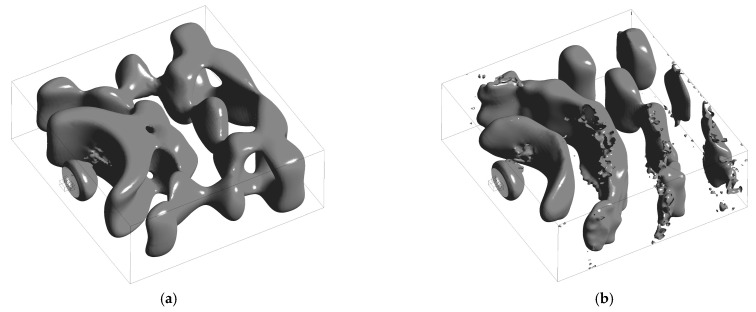
Iso-contours (*dp*/*dt* = 2 · 10^−4^ Pa/s, LES): (**a**) baseline; (**b**) serrations.

The iso-surfaces (Figure 9) provide a three-dimensional view of the unsteady structures. In the baseline case, the *dp*/*dt* field forms large, contiguous lobes that extend over several passages, suggestive of spanwise-correlated loading. In the serrated case, the same threshold (*dp*/*dt* = 2·10^−4^ Pa/s) yields narrower, broken-up lobes that are segmented by the serration influence, with noticeably reduced lateral continuity. This topological change is consistent with serration-induced phase variation at the leading edge: the impinging turbulent eddies are decorrelated in the spanwise direction, which limits the constructive accumulation of unsteady forces and promotes more rapid downstream decay of the energetic structures. The qualitative differences between iso-surfaces Figure 9a,b thus reinforce the spectral evidence reported earlier—namely, that interaction noise content is governed by near-blade dynamics and is mitigated when spanwise coherence is disrupted.

The integrated aerodynamic loads reinforce this picture (Table 1). For all models, the serrated configuration carries higher axial (X) and lift-like (Y) loadings than the baseline (e.g., SA and k−ω SST show an X-force of roughly 1.9–2.4 N and Y-force of ~4–5.3 N), and more modest in SAS/LES. This systematic uplift in load is compatible with the pressure distribution: stronger LE pressure gradients and spanwise modulation around the serration geometry translate into enhanced unsteady loading, which is precisely the mechanism expected to feed the measured interaction noise band. The fact that SAS and LES predict smaller load increments aligns with their tendency to distribute energy over a broader range of resolved scales, consistent with the closer spectral agreement observed in the 2–5 kHz region.

The magnitude-squared coherence *γ*^2^ ∈ [0, 1] directly measures the fraction of linearly correlated fluctuations shared by two locations at a given frequency. Values close to 1 indicate strongly coherent, phase-consistent content, whereas values close to 0 indicate decorrelated fluctuations or content dominated by uncorrelated turbulence. Pressure fluctuations were recorded at 11 surface probes along the leading edge for both the baseline and serrated configurations (shown schematically in Figure 4).

**Figure 10:**
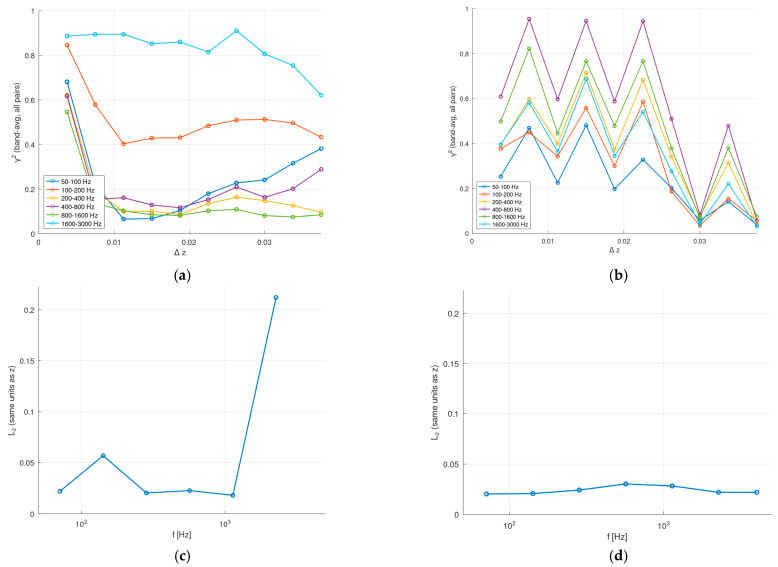
Spanwise coherence analysis: (**a**) band-averaged spanwise coherence, baseline; (**b**) band-averaged spanwise coherence, serrations; (**c**) coherence length *L_z_*, (fit from all pairs) baseline; (**d**) coherence length *L_z_* (fit from all pairs), serrations.

The extracted coherence length, *L_z_*, provides a single-number characterization of how quickly correlation decays along the span; a larger *L_z_* indicates spanwise-extended coherent structures, while a smaller *L_z_* implies rapid decorrelation (Figure 10c,d). In practical terms, *L_z_* can be interpreted as a descriptor for the spanwise “source size” over which unsteady loading may add constructively and thus contribute efficiently to far-field radiation; in turn, a reduced *L_z_* is consistent with weakened spanwise content and reduced potential for coherent summation. In the baseline configuration, the generally low coherence levels suggest that the leading edge pressure field is dominated by locally generated, weakly correlated turbulent structures rather than by spanwise-extended features. In contrast, the serrated leading edge introduces a geometric modulation that can redistribute coherence: while it is intended to reduce broadband coherence of the incoming interaction, it may also imprint coherence features at specific spanwise scales associated with the serration wavelength, which can appear as localized peaks and a more repeatable fitted *L_z_* (as also suggested in [46] by plotting the coherence parameter for various LE treatments). When coherence decays rapidly and remains close to zero over most spanwise separations, the fitted coherence length becomes sensitive to statistical fluctuations. This behavior is therefore interpreted as an indicator of weak spanwise organization rather than as a numerical artifact.

The results shown in Figure 10 were obtained by post-processing the simulated pressure signals with an in-house GNU Octave code (v9.2.0), following the procedure described in the Section 2. Each dataset covers approximately 0.5 s of simulated time (about 5000 samples at *Δt* = 1 × 10^−4^ s). For each probe location along the leading edge, the pressure time series was first converted to a fluctuating signal by removing its temporal mean and applying linear detrending. The auto- and cross-power spectral densities (PSD/CPSD) were then computed using Welch’s method (also called the averaged periodogram method, with Hanning windowing, 50% overlap, NFFT = 512). The magnitude-squared coherence was then evaluated for all probe pairs from these spectra. To improve statistical robustness, coherence values were band-averaged over predefined frequency intervals and further aggregated across all probes sharing the same spanwise separation *Δz* using median statistics. This procedure reduces the sensitivity to narrowband spectral peaks, numerical noise, and spatial bias associated with a fixed reference location, enabling a consistent comparison between the baseline and serrated leading edge cases.

## 4. Discussion

The observed differences between turbulence models can be interpreted in terms of their respective treatment of unsteady scales and eddy viscosity adaptation. Models such as k−ω SST tend to retain higher modeled turbulence levels near the leading edge, which explains their elevated low-to-mid frequency content. In contrast, SAS dynamically reduces eddy viscosity in regions of resolved unsteadiness, allowing partial scale resolution and a redistribution of energy toward higher frequencies. LES further extends this trend by resolving a broader portion of the turbulent spectrum, although at a significantly higher computational cost. These distinctions clarify why models may converge in the interaction noise band (1–3 kHz) while diverging outside of it.

For the baseline case at the 10° receiver position (Figure 6a), temporal resolution remains the decisive factor. The coarse time step (LES, *Δt* = 10^−4^ s) produces a noisy, poorly converged spectrum with spurious elevations around the 0.5–1.5 kHz band, whereas refining to *Δt* = 10^−5^ s regularizes the spectrum and brings LES closer to the experimental interaction noise slope beyond 1–2 kHz. Under the same *Δt*, SAS and k−ω SST offer comparable fidelity in the mid–high band; SAS tends to run slightly high in the few-hundred-hertz region and slightly low above ~5 kHz, while k−ω SST sits marginally under the experiment below 1 kHz but tracks well toward 2–8 kHz. In short, the ranking is unchanged: with adequate *Δt*, LES does not systematically outperform SAS or k−ω SST for the band of interest.

For the serrated geometry at the 10° receiver position (Figure 6b), the models reproduce the broadband character with distinct biases by frequency. The k−ω SST model remains generally above the measurements in the 0.5–2 kHz interval, while Spalart–Allmaras follows the experimental decay more closely beyond ~2 kHz. SAS is elevated at low frequencies (<~700 Hz) and shows localized underestimation around ~1 kHz; LES aligns well in the midband once *Δt* is tightened, with small deficits at the highest frequencies. The complementary PSD view (Figure 6c) corroborates these tendencies: SAS concentrates more energy at low frequencies, k−ω SST exhibits a higher PSD plateau over 1–3 kHz, and LES/SA sit lower in that range, consistent with their closer SPL match above 2 kHz. At 90° (Figure 6d), the angular sensitivity is evident. The k−ω SST model underestimates the 0.6–1.5 kHz hump seen in the experiment but approaches the envelope beyond ~2 kHz; SAS carries excess low-frequency levels and introduces localized peaks not present in the data; SA tracks the mid–high-frequency decay reasonably well but retains isolated over- and undershoots. LES tends to underpredict the midband yet converges toward the experimental slope at higher frequencies. The PSD comparison at 90° (Figure 6e) confirms this picture: SAS places disproportionate energy at low frequencies, k−ω SST is comparatively elevated over ~3–10 kHz, and LES takes an intermediate position with the smallest midband overfit among the models.

Overall, the obtained SPL and PSD spectra reinforce the main conclusions: interaction noise content is captured by all unsteady models once *Δt* is sufficiently small (observed for other time steps but more time-consuming); SAS and k−ω SST provide the most consistent agreement in the 2–5 kHz band that governs jet–blade interaction, with angle-dependent nuances; LES approaches the correct envelope with refined *Δt* but does not deliver a systematic advantage for the present runtime envelope; and the spectral energy distributions (Figure 6c,e) explain the observed SPL biases—low-frequency excess for SAS, 1–3 kHz elevation for k−ω SST, and comparatively balanced midband energy for LES/SA at 10°, with shifts at 90° that justify multi-angle validation.

Table 1 reports instantaneous surface-integrated blade loads (representative sample from the unsteady simulations after ~2000 time steps), not time-averaged or RMS values; therefore, they are used only as a supporting indicator, while model ranking is based primarily on spectral agreement with the measurements. Taking LES as the reference for physical fidelity, SAS is the closest model in Table 1, with deviations relative to LES of +10.3%/−0.4% (baseline X/Y) and −2.4%/+4.4% (serrated X/Y), whereas SA and k−ω SST show larger deviations (up to ~31%). This is consistent with the expected behavior of turbulence closures for leading edge interaction noise problems: LES resolves the large energy-containing eddies that drive unsteady blade loading, while URANS eddy–viscosity models may smooth part of the inflow–blade interaction; SAS partially recovers resolved unsteady content and therefore remains closer to LES.

Regarding TKE values in regions of interest integrated on the blades, a slight increase in values compared to the reference was observed, exceeding 30%, and at the domains’ interface (exit from the blade area), these values averaged over the surface were double those of the reference in the case of serrations. The same setup can be leveraged for the following deeper—but fully complementary—analyses: wall pressure RMS maps on the blades and along the leading edge, spanwise wall pressure coherence *γ*^2^(*f*, *Δz*) to quantify the decoherence introduced by the serration pitch, and FFT-based spectra of the axial/transverse force fluctuations (*C*_*L*_′(*f*); *C*_*D*_′(*f*)) to connect energy peaks directly to the interaction noise band. These additions would further consolidate the link between near-blade pressure gradients, integrated loading, and far-field spectra, while keeping the central conclusion (leading edge serrations can be credibly assessed with numerically affordable models for early-stage design and down-selection).

The interaction noise band targeted in this work lies in the kilohertz range, where turbomachinery blades commonly have strong components and where design changes (such as leading edge serrations) are expected to alter the source coherence and effective radiating area. Therefore, reporting coherence metrics in 0.9–5 kHz, and highlighting 1.6–3 kHz as a BPF-adjacent band, provides a mechanism-oriented complement to SPL spectra. It should be noted that below 1000 Hz, the pressure fluctuates quite inconsistently in the reference case, although there is data in the literature suggesting a decoupling also for straight leading edges as the distance from the reference increases [46,51]. We expected a lower coherence between sources at the serration peak and root [46], as is the case in Figure 10b. In [52], it is noted that the flow regime also affects the correlation parameters, especially at Reynolds numbers close to 10^5^ (this issue being remedied at higher speeds).

Several tendencies emerge from the presented results. The presence of interaction noise above ~1 kHz is captured by all unsteady models, while model ranking is angle-dependent: at 10° (quasi-axial), SAS/k−ω SST track the experimental spectra well and SA often matches the high-frequency roll-off; at 90° laterally, k−ω SST is generally the closest, with SA and SAS showing localized peaks that the experiment does not consistently exhibit. Importantly, no persistent narrowband tones appear in the measurements for the serrated case, and the simulations reflect this broadband character when time integration is sufficiently resolved. Unsteady relevant parameters were plotted from representative LES cases (here at *Δt* = 10^−4^ s, t ≈ 50 ms) to visualize the fluctuating field build-up; they are not intended to replace the time-resolved spectral analysis used for quantitative validation. Nevertheless, the observed patterns—the concentration of *dp*/*dt* near the leading edge, the fragmented spanwise structure with the presence of serrations, and triggered faster downstream attenuation—are fully consistent with the SPL/PSD trends and with the mentioned aerodynamic loads. The same computational setup can be extended to time-averaged unsteady statistics (e.g., wall pressure RMS plots) and spanwise correlation along the leading edge; however, even in the present form the figures already support the central mechanism by which leading edge serrations (a bio-inspired solution) reduce the effective coherence of unsteady loading and, consequently, the broadband radiation.

Beyond the presented configuration, the main value of this work is the transferability of the validation workflow rather than a result tied to a single cascade. The same approach can be applied to other interaction noise problems—e.g., rotor–stator stages, fan/OGV systems, or compressor/turbine cascades—by preserving the key similarity parameters that govern the flow–blade mechanism (Mach and Reynolds numbers, incidence, and, most importantly, the relation between the dominant inflow length scales and the serration wavelength, together with a consistent Strouhal-based scaling of the frequency band of interest). In practice, the results provide a route for other studies: the use of a numerically affordable model (unsteady SAS or k−ω SST) with a time step sufficiently small to resolve the interaction band and to play with various serration designs/parameters and operating points/flow conditions; LES can be used for a limited set of cases where finer-scale content or detailed near-field statistics are required; coherence metrics (γ^2^ and the derived metrics such as coherence length) can contribute to verify that the intended mechanism—the reduced spanwise coherence of unsteady loading—is actually achieved. This combination makes it easier to down-select designs at laboratory scale and then carry the most promising candidates toward more representative turbomachinery geometries with reduced risk and fewer costly iterations.

## 5. Conclusions

The study shows that bio-inspired leading edge serrated cascades can be modeled with mid-cost turbulence closures, making routine broadband assessments practical. In the band where jet–blade interaction governs radiation (≈2–5 kHz), SAS and k−ω SST reproduce the experimental spectral slope and level with a consistency comparable to—and often indistinguishable from—LES under comparable runtime constraints. This means that the approach is not only feasible but operationally convenient: engineers can screen serrated designs without committing to the computational expense of full LES.

The near-blade region—unsteady separation and inflow–edge interaction—dominates radiation across most of the spectrum, while the outer-domain contribution decays rapidly with frequency, especially at the relatively low Reynolds number considered here. This explains why achieving accurate near-wall dynamics and temporal resolution leads to the largest gains in predictive fidelity and why the conclusions hold at both microphones despite their different perspectives.

A limitation of the current coherence implementation is that probe locations are described only by z; therefore, the analysis captures spanwise decorrelation but cannot isolate potential streamwise/chordwise effects introduced by local geometric features. Nevertheless, the reported metrics are sufficient to support design down-selection because they provide an interpretable, geometry-sensitive mechanism indicator in the same frequency ranges where the acoustic benefit is observed.

Taken together, the findings are relevant and encouraging for design practice. They show that leading edge serration concepts can be evaluated using computationally efficient turbulence models that reproduce the measured spectral trends in the interaction noise band, thereby enabling rapid design iteration and evidence-based down-selection. This study achieves consistent experiment–simulation agreement over the 2–5 kHz range, clarifies model performance with clear angular sensitivity, and confirms that the dominant acoustic sources are localized to the near-blade region. These results support the practical adoption of leading edge serrations in early-stage workflows and motivate targeted LES only when additional scale resolution is demonstrably required.

## Figures and Tables

**Table 1:** Aerodynamic loading on blades (instant averaged values on surface after ≈2000 time steps).

Model	Geometry	Force on X (Axial)	Force on Y (⟂ to Axis)
Spalart	serr	5.175 N	15.959 N
	bsln	2.738 N	11.908 N
k−ω SST	serr	4.845 N	16.616 N
	bsln	2.637 N	11.273 N
SAS	serr	4.505 N	15.298 N
	bsln	4.188 N	14.218 N
LES	serr	4.614 N	14.650 N
	bsln	3.797 N	14.275 N

## Data Availability

The codes used in the additional processing of data are available upon reasonable request.

## References

[1] Akiwate D.C., Parry A.B., Joseph P., Paruchuri C.C. On the balance between the tonal and broadband noise of uninstalled propellers. Proceedings of the AIAA Aviation 2021 Forum.

[2] Peeters B., Nusselder R. (2019). Overview of Critical Noise Values in the European Region, M + P Report Revision 4, Prepared for EPA Network Interest Group on Noise Abatement (IGNA). https://epanet.eea.europa.eu/reports-letters/reports-and-letters/ig-noise_critical-noise-values-in-eu.pdf.

[3] Salze E., Pereira A.A., Brandstetter C., Clair V., Gea-Aguilera F., Lamidel D., Marjono J., Buszyk M., Polacsek C., Maier R. Noise Reduction of Aero-Engines Using Innovative Stators with Leading Edge Features. Proceedings of the 30th AIAA/CEAS Aeroacoustics Conference.

[4] Du H., Jiang H., Yang Z., Xia H., Chen S., Wu J. (2024). Experimental Investigation of the Effect of Bio-Inspired Wavy Leading-Edges on Aerodynamic Performance and Flow Topologies of the Airfoil. Aerospace.

[5] Pochkin Y., Khaletskiy Y. (2015). Aircraft Fan Noise Reduction Technology Using Leaned Stator Blades. Procedia Eng..

[6] Wei Z., Wang S., Farris S., Chennuri N., Wang N., Shinsato S., Demir K., Horii M., Gu G.X. (2023). Nature-inspired three-dimensional surface serration topologies enable silent flight by suppressing airfoil-turbulence interaction noise. arXiv.

[7] Liu X., Zang B., Azarpeyvand M. (2022). Wake-aerofoil interaction noise control with trailing-edge serrations. Exp. Therm. Fluid Sci..

[8] Moreau S., Roger M. (2024). Turbomachinery Noise Review. Int. J. Turbomach. Propuls. Power.

[9] Kholodov P., Moreau S. (2020). Identification of Noise Sources in a Realistic Turbofan Rotor Using Large Eddy Simulation. Acoustics.

[10] Cican G., Frigioescu T.-F., Crunteanu D.-E., Cristea L. (2023). Micro Turbojet Engine Nozzle Ejector Impact on the Acoustic Emission, Thrust Force and Fuel Consumption Analysis. Aerospace.

[11] Lu T., Liu C., Wang N., Shao C., Li Y. (2025). Ultra-broadband acoustic metaliner for fan noise reduction. Int. J. Mech. Sci..

[12] Cattanei A., Zecchin F.M., Di Pasquali A., Lazari A. (2021). Effect of the uneven blade spacing on the noise annoyance of axial-flow fans and side channel blowers. Appl. Acoust..

[13] Wang Y., Zhao K., Lu X.-Y., Song Y.-B., Bennett G.J. (2019). Bio-Inspired Aerodynamic Noise Control: A Bibliographic Review. Appl. Sci..

[14] Qiao C., Ye X., Wu Y., Li C. (2025). Insight into the Impact of Blade Perforation on the Aerodynamics and Acoustics of a Two-Stage Variable-Pitch Axial Fan. Energies.

[15] Lahoz M., Nabhani A., Saemian M., Bergada J.M. (2024). Wind Turbine Enhancement via Active Flow Control Implementation. Appl. Sci..

[16] Parra H.G., Ceron H.D., Gomez W., Gaona E.E. (2023). Experimental Analysis of Bio-Inspired Vortex Generators on a Blade with S822 Airfoil. Energies.

[17] Wang J., Nakata T., Liu H. (2019). Development of Mixed Flow Fans with Bio-Inspired Grooves. Biomimetics.

[18] Juangphanich P. (2019). Bio-Inspired Design of a Turbine Stage. Ph.D. Thesis.

[19] Tan J., Dong P., Gao J., Wang C., Zhang L. (2023). Coupling Bionic Design and Numerical Simulation of the Wavy Leading-Edge and Seagull Airfoil of Axial Flow Blade for Air-conditioner. J. Appl. Fluid Mech..

[20] Rong J., Liu H. (2022). Effects of owl-inspired leading-edge serrations on tandem wing aeroacoustics. AIP Adv..

[21] Wang L., Liu X., Wu L., Li D. (2022). Effect of the asymmetric bio-inspired trailing-edge serrations on sound suppression in a coupled owl-based airfoil. Appl. Acoust..

[22] Xing Y., Chen W., Wang X., Tong F., Qiao W. (2023). Effect of Wavy Leading Edges on Airfoil Trailing-Edge Bluntness Noise. Aerospace.

[23] Li J., Liu C., Li X. (2021). Effects of Wavy Leading-Edge Protuberance on Hydrofoil Performance and Its Flow Mechanism. J. Mar. Sci. Eng..

[24] Wang D., Cai C., Zha R., Peng C., Feng X., Liang P., Meng K., Kou J., Maeda T., Li Q. (2024). Impact of Leading-Edge Tubercles on Airfoil Aerodynamic Performance and Flow Patterns at Different Reynolds Numbers. Energies.

[25] Pal A., Ghoshal R. (2024). Acoustic radiation characteristics of shark skin inspired surface modified plates. Sci. Rep..

[26] Cheng M., Zhu Z., Wu B., Ye L., Song K. (2024). Simulation and Experimental Study of the Suppression of Low-Frequency Flow Noise Signals by a Placoid-Scale Skin. Appl. Sci..

[27] Lloyd C.J., Peakall J., Burns A.D., Keevil G.M., Dorrell R.M., Wignall P.B., Fletcher T.M. (2021). Hydrodynamic efficiency in sharks: The combined role of riblets and denticles. Bioinspir. Biomim..

[28] Powerful Owl. Adaptations in the Grampians. https://biology-adaptations-telkington.weebly.com/powerful-owl.html.

[29] Arrondeau B., Rana Z.A. (2020). Computational Aerodynamics Analysis of Non-Symmetric Multi-Element Wing in Ground Effect with Humpback Whale Flipper Tubercles. Fluids.

[30] Day L. (2023). Sharkskin Coating Reduces Airliner Fuel Use, Emissions. Hackaday. https://hackaday.com/2023/08/23/sharkskin-coating-reduces-airliner-fuel-use-emissions/.

[31] Vathylakis A. (2015). Reduction of Broadband Trailing Edge Noise by Serrations. Doctoral Thesis.

[32] Lau A.S., Haeri S., Kim J.W. (2013). The effect of wavy leading edges on aerofoil–gust interaction noise. J. Sound Vib..

[33] Thompson C., Biler H., Symon S., Ganapathisubramani B. (2023). Effects of integral length scale variations on the stall characteristics of a wing at high free-stream turbulence conditions. J. Fluid Mech..

[34] Sivakumar A., Porteous R., Mimani A., Doolan C.J. An experimental investigation of turbulent boundary-layer interaction with different serrated trailing-edge configurations. Proceedings of the ACOUSTICS 2015, Annual Conference of the Australian Acoustical Society.

[35] Tian H., Lyu B. (2024). The impact of non-frozen turbulence on the modelling of the noise from serrated trailing edges. J. Fluid Mech..

[36] Moreau S., Bampanis G., Roger M. Analytical and experimental investigation of leading-edge noise reduction on a flat plate with serrations. Proceedings of the AIAA AVIATION 2020 FORUM.

[37] Huang X. (2019). A theoretical study of serrated leading edges in aerofoil and vortical gust interaction noise. Adv. Aerodyn..

[38] Ayton L.J., Kim J.W. (2018). An analytic solution for the noise generated by gust–aerofoil interaction for plates with serrated leading edges. J. Fluid Mech..

[39] Tong F., Qiao W., Chen W., Cheng H., Wei R., Wang X. (2018). Numerical analysis of broadband noise reduction with wavy leading edge. Chin. J. Aeronaut..

[40] Kaya M.N., Satcunanathan S., Meinke M., Schröder W. (2025). Leading-Edge Noise Mitigation on a Rod–Airfoil Configuration Using Regular and Irregular Leading-Edge Serrations. Appl. Sci..

[41] Ajayi O.O., Unser L., Ojo J.O. (2023). Implicit rule for the application of the 2-parameters RANS turbulence models to solve flow problems around wind turbine rotor profiles. Clean. Eng. Technol..

[42] Ali I., Hussain T., Unar I.N., Kumar L., Ahad I.U. (2024). Turbulence model study for aerodynamic analysis of the leading edge tubercle wing for low Reynolds number flows. Heliyon.

[43] Muhammed M., Virk M.S. (2024). On the Fidelity of RANS-Based Turbulence Models in Modeling the Laminar Separation Bubble and Ice-Induced Separation Bubble at Low Reynolds Numbers on Unmanned Aerial Vehicle Airfoil. Drones.

[44] (2012). Acoustics—Determination of Sound Power Levels and Sound Energy Levels of Noise Sources Using Sound Pressure—Precision Methods for Anechoic Rooms and Hemi-Anechoic Rooms.

[45] Totu A.-G., Deaconu M., Cristea L., Bogoi A., Crunțeanu D.-E., Cican G. (2024). Experimental Analysis of Acoustic Spectra for Leading/Trailing-Edge Serrated Blades in Cascade Configuration. Processes.

[46] Teruna C., Avallone F., Casalino D., Ragni D. (2020). Numerical Investigation of Leading Edge Noise Reduction on a Rod-Airfoil Configuration Using Porous Materials and Serrations. J. Sound Vib..

[47] Jiang R.P., Liu P.Q., Zhang J., Guo H. (2024). Numerical Modal Analysis of the Correlation Between Spanwise Vortex Shedding and Far-field Aeolian Noise for Square and Circular Wall-mounted Cylinders. J. Appl. Fluid Mech..

[48] Celik A., Mayer Y., Azarpeyvand M. (2021). On the aeroacoustic characterization of a robust trailing-edge serration. Phys. Fluids.

[49] Letica S., Alexander W.N. (2021). Understanding the Impact of a Serrated Trailing Edge on the Unsteady Hydrodynamic Field. J. Aerosp. Eng..

[50] Van Der Velden W.C.P., Van Zuijlen A.H., De Jong A.T., Bijl H. (2015). Estimation of Spanwise Pressure Coherence Under a Turbulent Boundary Layer. AIAA J..

[51] Hu N. (2021). Coherence of wall pressure fluctuations in zero and adverse pressure gradients. J. Sound Vib..

[52] Dos Santos F.L., Botero-Bolivar L., Venner C.H., De Santana L.D. (2023). Wall-pressure spectra, spanwise correlation, and far-field noise measurements of a NACA 0008 airfoil under uniform and turbulent inflows. Appl. Acoust..

